# CpG-island methylation study of liver fluke-related cholangiocarcinoma

**DOI:** 10.1038/bjc.2011.102

**Published:** 2011-03-29

**Authors:** R Sriraksa, C Zeller, M A El-Bahrawy, W Dai, J Daduang, P Jearanaikoon, S Chau-in, R Brown, T Limpaiboon

**Affiliations:** 1 Graduate School, Khon Kaen University, Khon Kaen 40002, Thailand; 2 Epigenetics Unit, Department of Surgery and Oncology, Hammersmith Hospital, Imperial College, Du Cane Road, London W12 0NN, UK; 3 Department of Histopathology, Hammersmith Hospital, Imperial College, Du Cane Road, London W12 0NN, UK; 4 Centre for Research and Development of Medical Diagnostic Laboratories, Faculty of Associated Medical Sciences, Khon Kaen University, Khon Kaen 40002, Thailand; 5 Department of Surgery, Liver fluke and Cholangiocarcinoma Research Center, Faculty of Medicine, Khon Kaen University, Khon Kaen 40002, Thailand

**Keywords:** DNA methylation, cholangiocarcinoma, liver fluke, OPCML, DcR1, biomarkers

## Abstract

**Background:**

Genetic changes have been widely reported in association with cholangiocarcinoma (CCA), while epigenetic changes are poorly characterised. We aimed to further evaluate CpG-island hypermethylation in CCA at candidate loci, which may have potential as diagnostic or prognostic biomarkers.

**Methods:**

We analysed methylation of 26 CpG-islands in 102 liver fluke related-CCA and 29 adjacent normal samples using methylation-specific PCR (MSP). Methylation of interest loci was confirmed using pyrosequencing and/or combined bisulfite restriction analysis, and protein expression by immunohistochemistry.

**Results:**

A number of CpG-islands (*OPCML*, *SFRP1*, *HIC1*, *PTEN* and *DcR1*) showed frequency of hypermethylation in >28% of CCA, but not adjacent normal tissues. The results showed that 91% of CCA were methylated in at least one CpG-island. The *OPCML* was the most frequently methylated locus (72.5%) and was more frequently methylated in less differentiated CCA. Patients with methylated *DcR1* had significantly longer overall survival (Median; 41.7 *vs* 21.7 weeks, *P*=0.027). Low-protein expression was found in >70% of CCA with methylation of *OPCML* or *DcR1*.

**Conclusion:**

Aberrant hypermethylation of certain loci is a common event in liver fluke-related CCA and may potentially contribute to cholangiocarcinogenesis. The *OPCML* and *DcR1* might serve as methylation biomarkers in CCA that can be readily examined by MSP.

Cholangiocarcinoma (CCA), a malignancy originating from biliary epithelium, has been considered as a rare disease, which globally accounts for 10–25% of primary liver cancers (Kamangar *et al*, 2006). As the incidence and mortality rates for intrahepatic CCA (ICC) have markedly increased worldwide (Patel, 2002; Khan *et al*, 2005), there has been growing interest in this cancer. Cholangiocarcinoma represents a major public health problem in Thailand, particularly in the northeast region where *Opisthorchis viverrini* (OV) infection remains highly endemic. The prevalence of OV infection in this region is 15.7%, while the average in Khon Kaen (the centre for CCA collection in the present study) is 24.5% (Sripa *et al*, 2007). Here, CCA accounts for the highest incidence worldwide with truncated age-standardised incidence of ages >35 years up to 317.6 per 100 000 population (Sriamporn *et al*, 2004). Infection with OV occurs when raw or undercooked cyprinoid fish with the infective stage of metacercariae is ingested (Sripa *et al*, 2007). However, OV infection alone does not cause CCA. Animal experiments suggest that OV infection in combination with nitrosamine administration is the causation of CCA in a dose-dependent manner (Thamavit *et al*, 1978, 1987). Honjo *et al* (2005) conducted a population-based case–control study on genetic and environmental risk factors for OV-related CCA. They showed that anti-OV antibody ⩾0.200 was the strongest risk indicator. Furthermore, dietary habits during the past 10 years, raw fish intake (OV infection) and fermented fish or pork intake (exogenous nitrosamine) were significantly associated with an increased risk. Alcohol drinking, but not smoking showed a higher risk in which alcohol may affect metabolic pathways of endogenous and exogenous nitrosamines. The prognosis of CCA is generally poor with <5% of patients surviving >5 years and has not significantly improved over the past three decades (Shaib and El-Serag, 2004). This can partly be attributed to the fact that CCA is difficult to diagnose and often presents at a late clinical stage. Biomarkers, which allow earlier detection of CCA could potentially impact on patient survival by allowing earlier treatment. Although surgical resection can be potentially curative, only a few patients are suitable for surgery because of spread of the disease when late presenting, and effective non-surgical therapies are limited (Patel, 2006).

Epidemiological and experimental evidence implicate the carcinogenic liver fluke as a major risk factor of CCA. It is possible that chronic inflammation driven by the infection makes the biliary epithelium more susceptible to neoplastic transformation. (Thamavit *et al*, 1978; IARC, 1994). However, the exact molecular events involved in cholangiocarcinogenesis are not well understood. Like other tumours, CCA is a result of a multistep process in which genetic and epigenetic aberrations of regulatory genes are accumulated. For instance, genetic alterations of *K-ras* (Ohashi *et al*, 1995) and *p53* (Limpaiboon *et al*, 2002; Liu *et al*, 2006b) have frequently been reported in CCA. Aberrant DNA methylation at CpG-islands associated with transcriptional silencing is widely observed in cancers (Esteller, 2006). DNA methylation is a potential rich source of diagnostic, prognostic or predictive biomarkers of clinical outcome. Hypermethylation in CCA has been reported in tumour suppressor genes related to cell cycle (*p16*, *14–3–3σ*, *p73, p14*, *p15*), apoptosis (*DAPK, SEMA3B*) and cell adhesion (*APC, E-cadherin, TIMP3*, *THBS1*) (Lee *et al*, 2002; Tannapfel *et al*, 2002; Tischoff *et al*, 2005; Yang *et al*, 2005; Chinnasri *et al*, 2009). Furthermore, several reports demonstrated hypermethylation of genes involved in DNA repair and carcinogen metabolism, such as *hMLH1, MGMT* and *GSTP1* (Lee *et al*, 2002; Limpaiboon *et al*, 2005; Yang *et al*, 2005). However, while these previous studies have reported aberrant methylation at several loci in CCA, their evaluation as diagnostic or prognostic biomarkers is still limited.

To further elucidate the role of methylation in liver fluke-related CCA, we here analysed the methylation status by methylation-specific PCR (MSP) of a defined set of 26 CpG islands at genes, which function in cell cycle, apoptosis, DNA repair and cell–cell interaction, which have been previously reported in CCA and other types of cancer as frequently methylated. Methylation-specific PCR was performed in 102 primary CCA and where possible adjacent normal tissues. Methylation-specific PCR provides a relatively simple and cost-effective assay that can be used to assess methylation in clinical samples, however, for loci of interest, we have confirmed methylation status by bisulfite pyrosequencing or combined bisulfite restriction analysis (COBRA). We have identified differential hypermethylation of a number of CpG-islands and reported the associations to clinicopathological parameters.

## Materials and methods

### Samples and clinicopathological data

Frozen tissues of 102 primary CCA and 29 tumour-adjacent normal samples were used. Moreover, those CCA samples, which had paraffin-embedded sections were also used for immunohistochemistry. Clinicopathological data of CCA patients, including age at initial diagnosis, gender, gross types, histological grades and the overall survival time (excluding patients who died within 4 weeks after surgery) were also collected. All of the samples and data were kindly supplied by the Liver Fluke and Cholangiocarcinoma Research Centre, Khon Kaen University (Khon Kaen, Thailand). Among CCA samples, mean age at initial diagnosis was 55 years (45.2–64.8 years), and 68 males and 34 females were accounted. Cholangiocarcinoma samples were classified into three gross types; mass forming (MF, *n*=65), periductal infiltrating (PI, *n*=26) and intraductal growth (ID, *n*=11) type, and two histological grades; well differentiated (*n*=74) and less differentiated (included moderate and poor differentiation, *n*=25). Median overall survival time of CCA patients was 29 weeks. The study was reviewed and approved by the Khon Kaen University Ethics Committee for Human Research (HE510651).

### DNA extraction and bisulfite modification

Genomic DNA was extracted from frozen liver tissues using DNeasy Tissue Kit (Qiagen, Hercules, CA, USA) according to the manufacturer's protocols. Genomic DNA (1 *μ*g) from each sample was modified by sodium bisulfite as described by Herman *et al* (1996) with slight modification as previously reported (Sriraksa *et al*, 2010). The quality of bisulfite-converted DNA was verified by PCR amplification using *Calponin*-specific primer sets as previously described (Teodoridis *et al*, 2005; Sriraksa *et al*, 2010).

### Methylation-specific PCR (MSP)

Methylated-specific primers and PCR conditions of 22 CpG-islands for MSP were obtained from methprimerDB (http://medgen.ugent.be/methprimerdb/index.php). In addition, the methylated-specific primers and PCR conditions for *14–3–3σ, DcR2, DR4* and *DR5* were obtained from previous studies (Lee *et al*, 2002; van Noesel *et al*, 2002; Liu *et al*, 2008). Methylation-specific PCR (25 *μ*l per reaction) was performed on a PTC-100 Thermal cycle (MJ Research INC., Ramsey, MN, USA) as the appropriate PCR conditions for each locus. Methylation-positive sample was defined by comparing the MSP product to those of diluted *in vitro* methyltransferase-treated placental DNA (positive control) and the weak signal was disregarded.

### Pyrosequencing

Pyrosequencing of bisulfite modified DNA was used to validate methylation status of candidate loci, *OPCML and DcR1*, previously identified by MSP. The primers for *OPCML* amplification and sequencing were; 5′-GGGAGTGTGAGATGTATGTGAGTG-3′ (forward), 5′-biotin-TACCCCCAAAACCACAACTAATT-3′ (reverse) and 5′-AGAGGTAGGTTTGTTGTGT-3′ (sequencing) and for *DcR1*; 5′-TTGGTAGTGTAGTTGTGGGAATTTTT-3′ (forward), 5′-biotin-TCTATCCCCAAAATTCCCTAA-3′ (reverse) and 5′-GGTAGTGTAGTTGTGGGAA-3′ (sequencing). The schematics of these specific primers for pyrosequencing are shown in Supplementary Figure 1, upper and lower panels. DNA used as a template of PCR was bisulfite modified using EpiTect Bisulfite Kit (Qiagen, West Sussex, UK), and the annealing temperature and MgCl_2_ concentration for *OPCML and DcR1* were 63 °C, 2 mM and 61 °C, 3.6 mM, respectively. Pyrosequencing of PCR products was performed using PyroGold Reagent kit (Biotage, Uppsala, Sweden) according to the manufacturer's instruction. The methylation percentage at individual CpG sites was then analysed using the Pyro Q-CpG software (Biotage).

### Combined bisulfite restriction analysis (COBRA)

COBRA was performed to validate methylation status of *OPCML*, for which specific primers, amplification condition and restriction enzyme used were obtained from methprimerDB (http://medgen.ugent.be/methprimerdb/index.php). The positions of *OPCML*-specific primers for COBRA were located in the same region as for MSP (Supplementary Figure 1, upper panel). Digestion was performed by incubation at 37 °C for 2 h, before separation on a 2% agarose gel in which band intensity and methylation level were evaluated.

### Immunohistochemistry

The expression of OPCML and DcR1 was examined in formalin fixed paraffin-embedded tissue sections of primary CCA using immunohistochemistry as previously described (Hussain *et al*, 2010). Antigen retrieval was performed by microwave heating in EDTA (pH 9.0) for 20 min. To block non-specific antibody binding, sections were incubated with 2.5% normal horse serum (Vector Laboratories, Peterborough, UK) for 10 min. Here, sections were incubated overnight at 4 °C with OPCML monoclonal mouse antibody (1 : 1000 dilution; Anti-human OBCAM: MAB27771, R&D Systems, Abingdon, UK) or DcR1 monoclonal mouse antibody (1 : 100 dilution; DcR1 (L19): sc-73890, Santa Cruz Biotechnology, Heidelberg, Germany). Positive (breast carcinoma) and negative (PBS instead of primary antibody) controls were also conducted. After washing with phosphate-buffered saline (PBS) containing 0.1% Tween-20, sections were incubated with secondary antibody for 30 min at room temperature (ImmPRESS Universal Antibody Polymer Detection Kit, Vector Laboratories). Antigen–antibody reaction was visualised using diaminobenzidine chromogen and sections were counterstained with haematoxylin. All slides were examined and scored by two independent observers. The staining was scored as negative (=0), or positive with weak (=1), moderate (=2) and strong (=3) intensity.

### Statistical analysis

The statistical analysis was performed using SPSS software (SPSS version 16.0, Chicago, IL, USA). The correlation between methylation of each CpG-island and clinicopathological features of CCA patients including gender, gross types and histological grades, was analysed using *χ*^2^-test or Fisher's exact test. The correlation between methylation status and age at initial diagnosis was analysed using Mann–Whitney *U*-test. The association of methylation and overall survival time was determined using Kaplan–Meier method by log-rank test or univariate analysis by Cox regression. The association between MSP and pyrosequencing, COBRA or protein expression was analysed using *χ*^2^-test. Two sided *P*<0.05 was considered as statistically significant.

## Results

### Frequency of CpG-island methylation and concurrent methylation in cholangiocarcinoma

**Figure 1:**
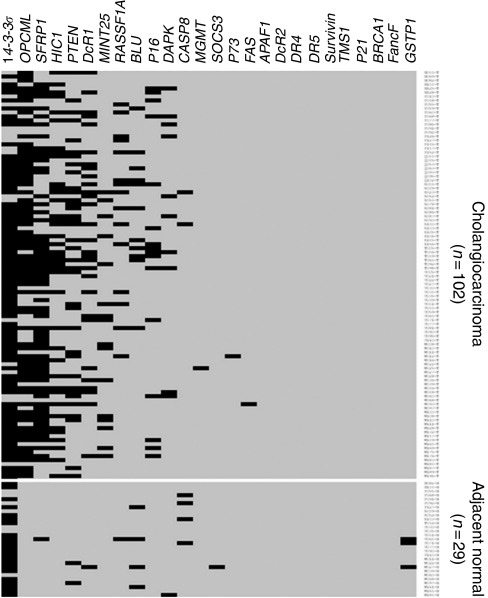
Methylation profile of 26 CpG-islands in cholangiocarcinoma and adjacent normal samples. Black and grey boxes represent positive and negative methylation by MSP, respectively.

**Figure 2:**
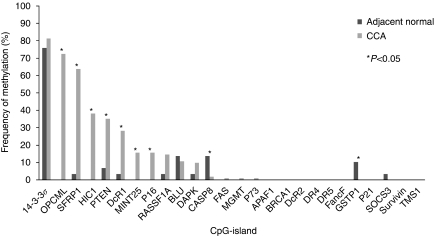
Histogram represented frequency of methylation of 26 CpG-islands in adjacent normal samples (*n*=29) and cholangiocarcinoma (*n*=102).

The CpG-island hypermethylation of 26 loci was investigated in 102 primary CCA and 29 matched adjacent normal tissues using MSP (Figures 1 and 2). All bisulfite-treated DNA samples were tested with *Calponin*-specific primer sets to demonstrate successful bisulfite modification before MSP analysis (Teodoridis *et al*, 2005; Sriraksa *et al*, 2010). The CpG-islands with high frequency of methylation in CCA included *14–3–3σ*, *OPCML*, *SFRP1*, *HIC1*, *PTEN* and *DcR1* (81.4, 72.5, 63.7, 38.2, 35.3 and 28.4%, respectively). Moderately frequent methylation was found for *MINT25*, *p16*, *RASSF1A* and *BLU* (15.7, 15.7, 14.7 and 10.8%, respectively) and low frequency was observed for *DAPK*, *CASP8*, *FAS*, *MGMT* and *p73* in 1–10% of CCA. No methylation was detected for *APAF1*, *BRCA1*, *DcR2*, *DR4, DR5, FancF*, *GSTP1*, *p21*, *SOCS3*, *Survivin* and *TMS1*. Adjacent normal tissues showed no or very low levels of methylation at all loci, with the exception of *14–3–3σ* for which a high frequency of methylation (75.9%) was found in normal adjacent tissue, and therefore methylation data for *14–3–3σ* has been excluded from subsequent analysis. After excluding *14–3–3σ*, 91% (93 out of 102) of CCA samples were methylated in at least one locus out of the 14 loci (*OPCML*, *SFRP1*, *HIC1*, *PTEN*, *DcR1*, *MINT25*, *p16*, *RASSF1A*, *BLU*, *DAPK*, *CASP8*, *FAS*, *MGMT* and *p73*).

**Figure 3:**
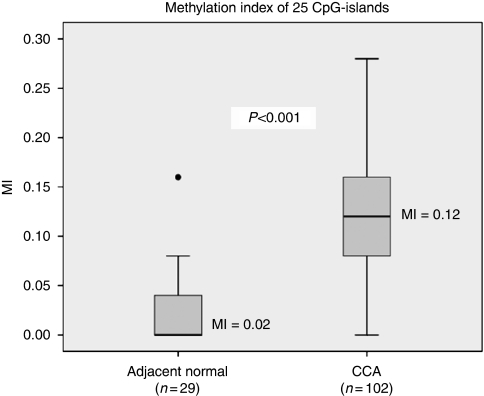
Methylation index (MI) between adjacent normal samples and CCA in which CCA samples showed significantly higher MI than adjacent normal tissues.

We analysed the tumours for a methylation index (MI), which was calculated from the ratio between the number of methylated CpG-islands and the number of analysed CpG-islands (Gutierrez *et al*, 2004). The average methylation index of CCA was significantly higher than that in adjacent normal tissues (average MI; 0.12 *vs* 0.02, *P*<0.001) as shown in Figure 3. Concordant methylation in CCA was found between pairs of CpG-island including *OPCML* and *SFRP1* (*P*=0.007), *OPCML* and *HIC1* (*<*0.001), and *HIC1* and *PTEN* (*P*=0.002). No significant association between other pairs of CpG-island was observed. In addition, we also analysed the association of MI and clinicopathological data of CCA patients. However, no significant correlation of clinical data and MI was observed.

### OPCML

The highest methylation frequency was observed in 72.5% of CCA for *OPCML*, whereas no methylation of this locus was revealed in adjacent normal samples. In addition, methylation of *OPCML* was found more frequently in less differentiated type (88% 22 out of 25) than in the well-differentiated types of CCA (67.6% 50 out of 74) (*P*=0.047). To confirm the methylation detected by MSP, an independent method of methylation analysis, COBRA, was used. The correlation between MSP and COBRA results was analysed using *χ*^2^-test and significant association with MSP was found at *OPCML* by COBRA (80.0% of tested samples, *P*=0.025). To address whether methylation has effect on gene expression, immunohistochemistry was performed to determine OPCML protein expression in CCA samples. Low or no OPCML protein expression was detected in CCA, 63% (58 out of 92) weakly expressed OPCML and 26% (24 out of 92) exhibited no detectable expression. Low-protein expression was found in 88% (59 out of 67) of *OPCML*-hypermethylated CCA, however, as the vast majority of tumours (89%) had no or very low OPCML expression, it was not feasible to statistically analyse the association between expression and methylation.

### DcR1

**Figure 4:**
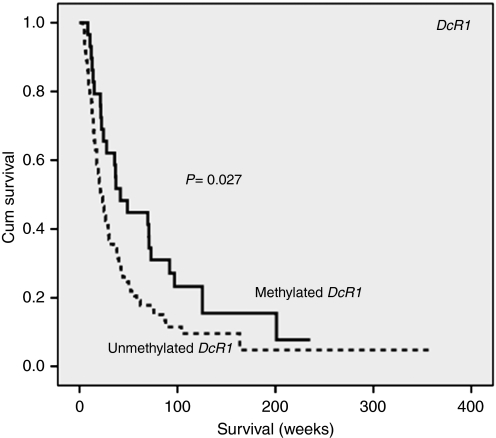
The association between methylation of *DcR1* and overall survival time of cholangiocarcinoma patients. Patients with methylated *DcR1* had longer overall survival time than those without (Kaplan–Meier analysis using log-rank test, *P*=0.027).

In the present study, *DcR1* CpG island methylation was observed in 28.4% of CCA. Interestingly, patients with methylated *DcR1* showed longer overall survival than those without (Median; 41.7 *vs* 21.7 weeks, *P*=0.027), Figure 4. The significance of methylated *DcR1* on survival raised the question on the role of methylation status of other *TRAIL*-related genes in CCA. To address this question, we next analysed methylation of CpG islands at *DcR2*, *DR4* and *DR5* in CCA samples, but no methylation was detected at these loci. To confirm the methylation detected by MSP, we showed a significant correlation between MSP and pyrosequencing in each individual CpG site of *DcR1* and also in the average of the four CpG sites examined. Moreover, a trend of correlation of methylation in CpG1 with patient survival was observed (*P*=0.08, data not shown), similar to that of MSP. Low or no DcR1 protein expression was detected in CCA, in which 54% (50 out of 92) showed low-DcR1 expression and 23% (22 out of 92) displayed no expression. Increased frequency of low-protein expression was also observed in 77% (20 out of 26) of *DcR1*-hypermethylated samples. In addition, low-DcR1 expression was observed in ID type (100% 11 out of 11), followed by MF (82.5% 47 out of 57) and PI types (58.3% 14 out of 24) (*P*=0.01).

## Discussion

In the present study, the majority of CCA (91%) shows aberrant methylation in at least one locus, suggesting that DNA methylation is a common event in CCA, which is in agreement with previous reports (Lee *et al*, 2002; Yang *et al*, 2005). The high-methylation frequency of *14–3–3σ* found in CCA and adjacent normal tissues is similar to that observed in normal breast tissue and breast cancer (Umbricht *et al*, 2001) suggesting that methylation of *14–3–3σ* is tissue specific regardless of pathological status. The high-methylation frequency presented for CpG islands at *OPCML*, *SFRP1*, *HIC1*, *PTEN* and *DcR1* suggests that many signalling pathways could be involved in the development and progression of CCA, although does not demonstrate that all are drivers of carcinogenesis. However, association of methylated *OPCML* and *DcR1* with patient clinicopathological data supports their role in tumour progression. The tumour suppressor gene *OPCML* (Opioid binding protein/cell adhesion molecule-like gene) belongs to the IgLON family of immunoglobulin (Ig) domain containing glycosylphosphatidylinositol (GPI)-anchored cell adhesion molecules that are involved in cell adhesion and cell–cell recognition (McNamee *et al*, 2002; Sellar *et al*, 2003). In addition, *OPCML* has been proposed as a stress- and p53-responsive gene, with the response impaired when the promoter becomes hypermethylated. Moreover, exogenous expression of OPCML in carcinoma cells lacking its expression leads to dramatic growth inhibition suggesting *OPCML* as a tumour suppressor (Sellar *et al*, 2003; Cui *et al*, 2008). The *OPCML* hypermethylation has been reported in several cancers for example, 33.3% of late stage ovarian cancer (Teodoridis *et al*, 2005), 70% of hepatocellular carcinoma (Liu *et al*, 2006a), 63.9% of invasive cervical cancer (Ye *et al*, 2008) and 57–100% of multiple carcinomas and lymphomas (Cui *et al*, 2008). We are the first to report *OPCML* methylation in CCA with high frequency (72.5%) with no methylation in normal adjacent tissue. The previous studies have demonstrated that CCA with less differentiation shows a poorer outcome and high incidence of metastases than well differentiated tumours (Nakajima *et al*, 1988; Nakanuma *et al*, 2000). The high incidence of *OPCML* methylation in CCA and its association with less differentiation together with its absence in normal tissue indicate that *OPCML* methylation could be used as an epigenetic biomarker for molecular prognosis and diagnosis of CCA.

The highly frequent methylation of *SFRP1*, *HIC1* and *PTEN* could lead to an increase in proliferation, inhibition of apoptosis and promotion of survival advantage in CCA through increased Wnt/*β*-catenin signal transduction through the impaired p53-responsive pathway and activated PI3K and its downstream effectors as previously reported in other cancers (Wales *et al*, 1995; Finch *et al*, 1997; Melkonyan *et al*, 1997; Besson *et al*, 1999; Nicoll *et al*, 2001; Sulis and Parsons, 2003; Chen *et al*, 2005; Manning and Cantley, 2007; Takagi *et al*, 2008; Fleuriel *et al*, 2009).

The defect of programmed cell death through p53-dependent pathway has been observed in CCA with mutational inactivation (41.6%) and LOH of p53 (32%) as shown in our previous studies together with p53 deacetylation because of *HIC1* methylation (38.2%) in this study. These phenomena impede p53 function, which allows cells to bypass apoptosis and survive DNA damage (Limpaiboon *et al*, 2002, 2004). It is surprising that death receptor-induced apoptosis was rarely methylated. These findings indicate the advantage for selective cancer treatment and good outcome in CCA patients. The decoy receptors have been postulated to account for TRAIL resistance as overexpression of *DcR1* and/or *DcR2* prevent cancer cells from TRAIL-induced apoptosis. Hypermethylation of *DcR1* (28.4%), but not death receptors (*DR4* and *DR5*) and other TRAIL signalling-related genes (*APAF1*, *CASP8* and *DcR2*) in our study indicates the potential use of recombinant TRAIL or TRAIL receptor agonistic monoclonal antibodies as selective anti-tumour therapy in CCA. To overcome TRAIL resistance that always happens, combined treatment with standard chemotherapeutics can enhance TRAIL-induced apoptosis. Previous studies showed correlation of methylated *DcR1* with improved prognosis in malignant mesothelioma and prostate cancer (Shivapurkar *et al*, 2004; Suzuki *et al*, 2006). As longer overall survival was observed in methylated *DcR1* patients, *DcR1* methylation may be useful as a prognostic marker of CCA.

In conclusion, promoter hypermethylation has been observed in many genes, which have important roles in carcinogenesis and progression of liver fluke-related CCA. Further study is warranted to validate the use of high-frequency methylated genes as potential biomarkers for diagnosis, prognosis and prediction of CCA patients. Moreover, tumour cells are significantly more sensitive to TRAIL-induced apoptosis than normal cells, thus the use of recombinant TRAIL or TRAIL receptor agonistic monoclonal antibodies for selective treatment of CCA in combination with chemotherapeutic drugs may improve patient survival.
