# Supplementary material for: CpG-island methylation study of liver fluke-related cholangiocarcinoma
Source: Br J Cancer. 2011 Mar 29;104(8):1313–8. doi: 10.1038/bjc.2011.102 (PMC3078588; doi:10.1038/bjc.2011.102)
Supplement: Supplementary Information [file bjc2011102x1.doc]

**Supplementary information**

**Supplementary Figure 1**


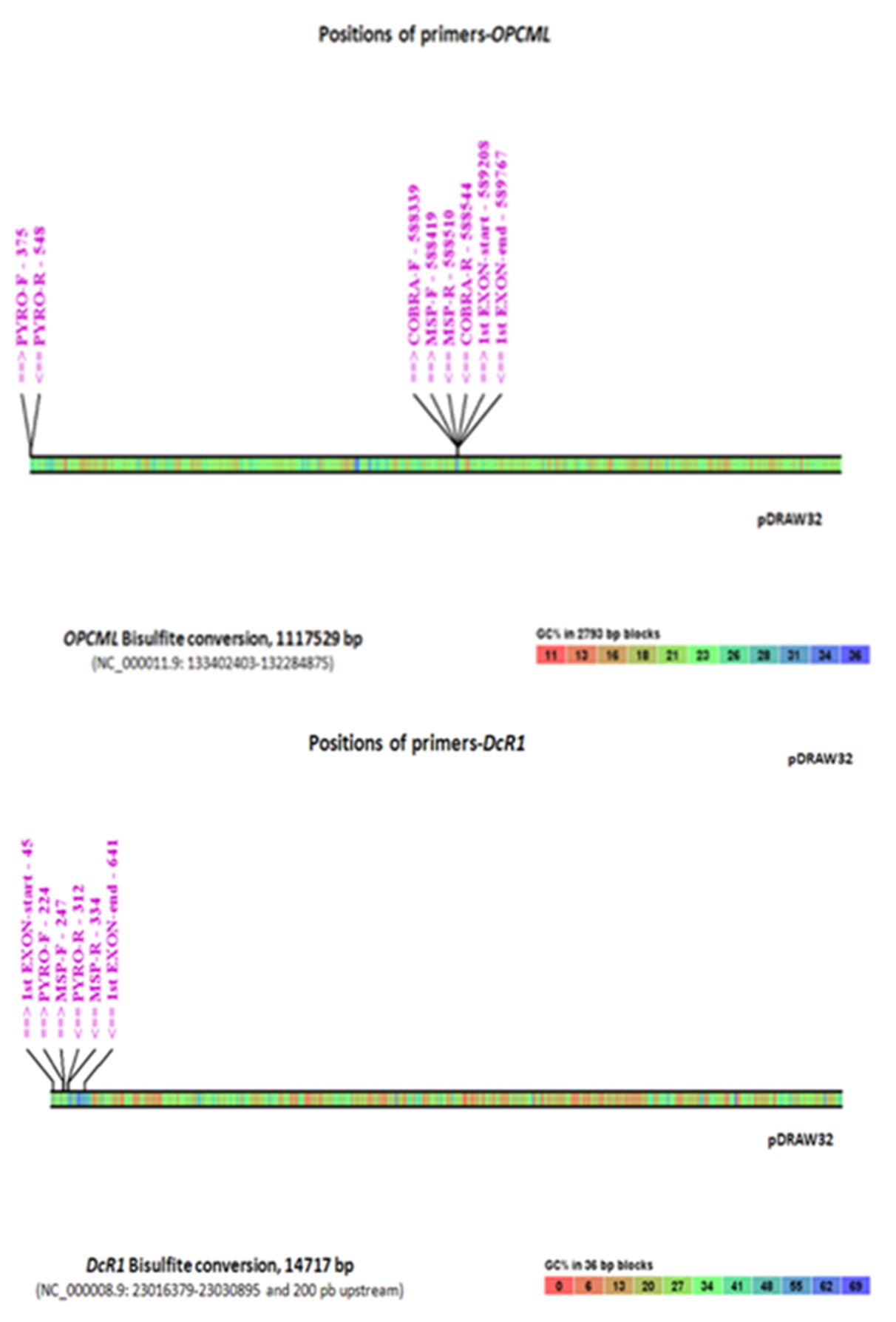


**Supplementary Figure 1** The schematics represent primer positions of MSP, pyrosequencing and COBRA for methylation study in *OPCML* (upper panel) and *DcR1* (lower panel) (pDRAW32 DNA analysis software).
